# Performance of HIV Prevention of Mother-To-Child Transmission Programs in Sub-Saharan Africa: Longitudinal Assessment of 64 Nevirapine-Based Programs Implemented in 25 Countries, 2000-2011

**DOI:** 10.1371/journal.pone.0130103

**Published:** 2015-06-22

**Authors:** Joël Ladner, Marie-Hélène Besson, Mariana Rodrigues, Joseph Saba, Etienne Audureau

**Affiliations:** 1 Rouen University Hospital, Epidemiology and Public Health Department, Rouen, France; 2 Axios International, 7 boulevard de la Madeleine, Paris, France; 3 Biostatistics and Epidemiology Unit, Paris Est University, hôpital Henri Mondor Hospital, Public Health, Assistance Publique Hôpitaux de Paris, Créteil, France; University of Washington, UNITED STATES

## Abstract

**Background:**

To evaluate the performance and to identify predictive factors of performance in prevention of mother-to-child HIV transmission programs (PMTCT) in sub-Saharan African countries.

**Methods:**

From 2000 to 2011, PMTCT programs included in the Viramune Donation Programme (VDP) were prospectively followed. Each institution included in the VDP provided data on program implementation, type of management institution, number of PMTCT sites, key programs outputs (HIV counseling and testing, NVP regimens received by mothers and newborns). Nevirapine Coverage Ratio (NCR), defined as the number of women who should have received nevirapine (observed HIV prevalence x number of women in antenatal care), was used to measure performance. Included programs were followed every six months through progress reports.

**Results:**

A total of 64 programs in 25 sub-Saharan African countries were included. The mean program follow-up was 48.0 months (SD = 24.5); 20,084,490 women attended in antenatal clinics were included. The overall mean NCR was 0.52 (SD = 0.25), with an increase from 0.37 to 0.57 between the first and last progress reports (p<.0001); NCR increased by 3.26% per year-program. Between the first and the last report, the number of women counseled and tested increased from 64.3% to 86.0% (p<.0001), the number of women post-counseled from 87.5% to 91.3% (p = 0.08). After mixed linear regression analysis, type of responsible institution, number of women attended in ANC, and program initiation in 2005-2006 were significant predictive factors associated with the NCR. The effect of the time period increased from earlier to later periods.

**Conclusion:**

A longitudinal assessment of large PMTCT programs shows that scaling-up of programs was increased in sub-Saharan African countries. The PMTCT coverage increased throughout the study period, especially after 2006. Performance may be better for programs with a small or medium number of women attended in ANC. Identification of factors that predict PMTCT program performance may help in the development and expansion of additional large PMTCT services in sub-Saharan Africa.

## Introduction

As home to more than two-thirds of the global population of individuals living with HIV/AIDS, sub-Saharan Africa has the highest HIV/AIDS burden of any region in the world. Of children newly infected with HIV in 2011, 90% lived in sub-Saharan Africa [1]. The very large majority of these infections were associated with mother-to-child transmission (MTCT). Effective interventions for the prevention of mother-to-child transmission (PMTCT) are critical to reducing the HIV/AIDS burden in sub-Saharan Africa.

The ability of antiretroviral (ARV) drugs to reduce the risk of MTCT of HIV was first demonstrated in the 1990s, and additional reductions in transmission rates have been achieved with increasingly effective ARV regimens, most notably with highly active antiretroviral therapy (HAART) [2–7]. PMTCT is critical to reducing the global HIV/AIDS burden in the developing world, and a variety of programs have provided more than 900,000 pregnant women with ARV prophylaxis or treatment by the end of 2012 [1]. According to a UNAIDS report, continued scale-up of PMTCT programs may make it possible to reach 90% of HIV+ pregnant women by 2015 [1]. In combination with other measures to reduce or eliminate vertical transmission of HIV, expansion of PMTCT services could help to reduce the number of newly infected children by 90%.

Because coverage of PMTCT programs throughout Africa is variable, many women do not have access to appropriate interventions in the antenatal care setting to prevent vertical transmission of HIV. While PMTCT programs have helped to reduce the rate of MTCT to less than 2% in industrialized countries, the coverage rate of effective antiretroviral regimens for PMTCT was only 57% in low- and middle-income countries in 2011 [8–12]. Additionally, in low- and middle-income countries, an estimated 35% of pregnant women were tested for HIV in 2010, and less than half (48%) of those living with HIV received the most effective regimens (excluding single-dose nevirapine [sd-NVP]) for preventing MTCT [13]. A systematic review found that uptake of counseling and testing among women attending antenatal care was relatively high, at 96% and 81% respectively, but that there was substantial attrition over time that resulted in lower percentages of women and infants receiving ARV prophylaxis [14].

Experiences from the field have identified a variety of barriers that limit the success of PMTCT programs in resource constrained settings, including management of the voluntary counseling and testing (VCT) process in antenatal clinics, drug accessibility and availability, infrastructure and health human resources, community-level issues, and individual-level issues related to socioeconomic status, education, psychological factors [15–21]. These barriers are not insurmountable, as suggested by findings that PMTCT programs that include rapid HIV testing and a structured process for assessing adherence to ARVs provided to HIV+ pregnant women and their newborn infants increases the number of women receiving these therapies [14].

Routine collection, analysis, and strategic use of data gathered throughout the delivery of PMTCT services is essential for reducing the incidence of HIV infections in children and improving the health of HIV-positive mothers [8]. Despite this need, recent reports demonstrate that there are limited data on which to base decisions related to improving access and adherence to PMTCT regimens or defining or evaluating contextual factors that affecting the program’s efficacy [22, 23]. In particular, longitudinal data with long follow-up are lacking to investigate the sustainability of programs and their potential ability to improve over time. While current strategies for PMTCT have evolved to include more complex ARV regimens, longitudinal analysis of the performance of PTMCT programs that used sd-NVP may be useful in identifying programmatic approaches and innovations that correlate with improved program performance [24]. Lessons learned from the experiences of large programs that include long-term follow-up may be useful for defining the parameters of future efforts to expand and improve PMTCT services in low- and middle-income countries.

The objectives of the study were to evaluate the overall performance of sd-NVP-based PMTCT programs and to identify predictive factors associated with the performance of PMTCT programs in sub-Saharan Africa countries.

## Methods

### Setting

Boehringer Ingelheim, the manufacturer of Viramune (nevirapine, sd-NVP), has offered the drug to developing countries since 2000 through the Viramune Donation Programme (VDP). Axios International has been responsible for sd-NVP management and monitoring of participating PMTCT programs [25].

Aggregate monitoring data collected from each program within the framework of the VDP were used. In the VDP, sd-NVP was provided free of charge to low-income countries for use in the PMTCT, and all sub-Saharan African countries were eligible to participate. The sd-NVP donation was available to governments directly through the Ministry of Health (MoH). Non-Governmental Organizations (NGOs) and International Agencies (IAs) were also eligible for the VDP if their PMTCT programs had approval from the government of the country where the programs were to be implemented. NGOs operated independently of the government, and more than 80% of NGOs were national organizations. International Agencies (IAs) were institutional multilateral partners, mostly agencies from the United Nations. Methods used for the collection of program-, and country-level data from countries have previously been reported [25]. The study was reviewed by the Ethics Committee (Comité de Protection des Personnes Nord Ouest 1 in France) reporting that a statement of Ethics Committee was not necessary.

### Program inclusion procedures

Institutions interested in participating in the VDP submitted a completed application form, which collected standardized information about the planned PMTCT program, including: country, type of responsible institution or organization, and the number of operational sites (i.e. health centers facilities) where the PMTCT program was to be implemented. Two independent experts reviewed submitted forms and made recommendations as to whether or not the submitting institution should be included the VDP. After expert approval, the sd-NVP was shipped to the institutions.

### Program follow-up procedures

According to the terms and conditions of the VDP, included responsible institutions were required to submit a standardized progress report every six months after their inclusion in the program. This progress report documented the number of women who registered in antenatal clinics (ANC), underwent pre-HIV test counseling and HIV testing, were HIV+ (which determined the HIV seroprevalence within the area that the program served) and received post-test counseling. Data were also collected on the number of women and infants receiving sd-NVP (one 200 mg tablet taken by the mother at the onset of the labor and an infant dose of 2 mg/kg [0.6 ml] of syrup to be taken within 72 hours after delivery) and the number of new PMTCT sites opened since the prior report.

### Process indicators

A PMTCT coverage indicator was calculated in order to assess the overall performance of each program. This indicator, the NVP coverage ratio (NCR), was defined as the observed number of women receiving sd-NVP divided by the expected number of women who should have received sd-NVP. This denominator was calculated as the number of women attending ANC visits multiplied by the observed HIV seroprevalence [25].

The proportion of women attending an ANC who participated in specific components PMTCT services (acceptance of pre-test counseling and HIV testing by pregnant women attending an ANC, post-test counseling, observed HIV prevalence, and receipt of sd-NVP by women and infants) was also calculated.

### Statistical methods

Descriptive statistics are given as means with their standard deviation (SD) for continuous variables and percentages for categorical variables. For each program, the cumulative incidence of follow-up in program-months was calculated. In order to assess the performance of the included institutions, univariate analysis was performed to identify potential predictors of the final NCR and the intermediate process indicators (women accepting pre-testing counseling, women tested, women counseled after testing, and women receiving sd-NVP). To do so, average numbers computed for the whole study period for each program were used. Anova or non parametric Kruskall-Wallis tests were used for comparisons of continuous data, depending on the normality of variable distribution; the following factors were considered: time period of the first progress report (2001–2002; 2003–2004; 2005–2006), type of responsible institution (government, NGO, international agency), mean number of PMTCT sites (0–20; 20–100; >100), and mean number of women attended an ANC per month (0–30; 30–100; >100). For the indicators of PMTCT services, the mean difference between first and last progress report was assessed. We used multivariate linear mixed models to assess the independent effect of each predictor of the NCR entered as the dependent variable, using all individual progress reports available instead of aggregating data. The institution was modeled as a random effect so as to account for the longitudinal structure of the data based on repeated measures. Candidate predictors for multivariate analysis were defined a priori, based on the hypothesized relationships to be tested. In addition to the predictors previously described, time since program initiation was entered as an independent variable and interactions were tested between this latter factor and the other predictors, in order to check for varying temporal evolutions according to the predictors. Beta coefficients are given with their 95% confidence intervals (CI), indicating the adjusted difference in NCR (0–100) between categories for qualitative variables or per 1-point difference in predictor value for continuous variables. A two-tailed p value <0.05 was considered to be significant. Statistical analyses were performed using Stata 11.0 software package (StatCorp, TX, USA).

### Role of funding source

There was no external source of funding for this research. Axios International is a commercial company, which has been responsible for managing and monitoring the Viramune Donation Program and distributing nevirapine (Viramune). Boehringer Ingelheim contracted the services of Axios International to manage the VDP. Boehringer Ingelheim had no role in study design, data collection, data analysis, data interpretation, or manuscript development. The corresponding author had full access to all data in the study and had final responsibility for the decision to submit for publication. All data analyses were conducted under the authorization from Boehringer Ingelheim.

## Results

From 2000 to 2011, a total of 64 programs in 25 sub-Saharan African countries were implemented under the VDP and described in Table 1. Thirty programs (46.9%) were managed by governmental institutions, primarily by MoH, mostly through their national AIDS control programs, 27 programs (42.2%) were managed by NGOs and seven programs (10.9%) managed by an international agency (IA) (Table 1). The mean total number of PMTCT sites per program was 360.4 (SD = 577.6, Median [M] = 111.5, Range [R] = 1–2,604).

**Table 1:** Number of programs, countries, responsible institutions, number of women attended in ante-natal clinics and PMTCT sites and median follow-up of the 64 programs included in the Viramune Donation Programme, sub-Saharan Africa, 2000–2011.

Countries	Number of programs included	Responsible institutions			Total of ANC visits	Total number of PMTCT sites	Median follow-up of months
		Govt	NGO	IA			
Benin	1	0	1	0	3,685	9	63.7
Botswana	1	1	0	0	66,673	1,069	15.2
Burkina Faso	2	1	1	0	75,592	63	48.3
Cameroon	5	1	3	1	543,890	816	40.5
CAR	1	1	0	0	64,186	105	67.6
CDR	5	0	4	1	965,053	291	39.9
Cote d’Ivoire	1	1	0	0	1,339,098	497	56.3
Ethiopia	2	2	0	0	1,917,074	1,295	54.1
Gabon	2	2	0	0	43,476	12	17.2
Ghana	2	1	1	0	130,111	23	42.7
Kenya	11	4	6	1	3,359,468	1,137	43.7
Lesotho	1	1	0	0	18,154	75	29.4
Malawi	2	0	1	1	1,359,511	106	62.1
Mali	1	1	0	0	92,079	121	24.2
Mozambique	2	0	2	0	411,839	177	37.0
Namibia	1	1	0	0	104,297	351	53.1
Nigeria	5	3	1	1	331,729	101	43.2
Rwanda	1	1	0	0	1,172,935	2,084	74.9
South Africa	4	4	0	0	2,760,197	3,388	55.4
Swaziland	1	1	0	0	62,261	248	81.0
Tanzania	5	1	4	0	782,126	587	42.6
Togo	2	1	1	0	83,676	87	28.3
Uganda	2	1	1	0	2,984,954	3,101	100.3
Zambia	3	0	1	2	760,266	484	72.6
Zimbabwe	1	1	0	0	639,562	9,519	45.6
Total	64	30	27	7	20,084,490	25,746	45.8

Govt: governments. IA: international agencies. NGOs: non-governmental organizations. ANC: Ante Natal Clinic. CDR: Congo Democratic Republic. CAR: Central African Republic.

Between the date of initial inclusion in the VDP and submission of the final progress report, for the 64 programs, the mean program follow-up was 48.0 months (SD = 24.5, M = 45.8, R = 17.2–100.3) (Table 1). Mean follow-up was 48.2 months (SD = 25.9, M = 45.6) in the 30 programs managed by governmental institutions, 41.7 months (SD = 18.9, M = 41.3) in the 27 programs implemented by NGOs, and 71.5 months (SD = 26.3, M = 61.0) in the seven programs managed by international agencies (p = 0.01).

During the study period, the mean number of women attending an ANC per program-month was 5,493 (SD = 7,349, M = 2,196). The mean number of women attending an ANC per program-month varied among programs managed by institutions: 7,659 (SD = 9,316, M = 2,645) for programs managed by governmental institutions, 3,194 (SD = 3,533, M = 1,580) for NGOs and 5,076 (SD = 6,784, M = 3.30) for IAs (p = 0.07).

Table 2 describes the evolution of PMTCT services indicators over the study period. The overall mean NCR was 0.52 (SD = 0.25, M = 0.52, R = 0.08–1.0), which increased significantly between the first and last progress report, from 0.37 for the first progress report to 0.57 for the last progress report (p < .0001) (Table 2). The NCR also varied significantly according to the type of responsible institution, with the highest NCR value for NGOs, and also according to the mean number of women attending in ANC (Table 2). Mean HIV prevalence was 9.9% (SD = 8.6) over the study period, and decreased not significantly between the first and the last progress report (Table 2).

**Table 2:** Results over the study period of antenatal voluntary counselling and testing, NVP access and Nevirapine coverage ratio (NCR) for the NVP-based programs according to African regions, responsible institutions, number of PMTCT sites and (N = 64 programs).

	Pre-counselled and HIV-tested	Post-test counselled*	HIV prevalence	Women sd-NVP received^†^	Newborns sd-NVP received^†^	Mean NCR (SD)
Responsible institutions						
Governments	67.2	63.5	12.3	61.3	48.9	0.41 (0.21)
NGOs	84.2	68.6	7	70.6	56.9	0.62 (0.24)
IAs	89.4	76.1	10.3	65.7	47.7	0.58 (0.26)
*p-value*	*0*.*001*	*0*.*65*	*0*.*06*	*0*.*35*	*0*.*31*	*0*.*006*
Mean number of women attended in ANC per month						
<30 (n = 19)	77.7	68.9	14.6	71.4	63.4	0.59 (0.30)
30 to 100 (n = 26)	83.9	67.1	9.1	66.1	49.4	0.58 (0.26)
>100 (n = 19)	64.2	65.4	6.2	59.5	46.7	0.34 (0.19)
*p-value*	*0*.*01*	*0*.*95*	*0*.*01*	*0*.*31*	*0*.*03*	*0*.*001*
Mean number of PMTCT sites						
<20 (n = 17)	73.4	78.0	6.4	64.5	49	0.46 (0.20)
20 to 100 (n = 16)	79.7	65.0	12.1	62.5	50.4	0.53 (0.25)
>100 (n = 31)	76.7	48.2	12.3	70.5	59.0	0.57 (0.27)
*p-value*	*0*.*89*	*0*.*01*	*0*.*03*	*0*.*37*	*0*.*24*	*0*.*32*
Time period of the first progress report						
2001–2002	74.9	73.2	14.2	61.0	46.1	0.45 (0.23)
2003–2004	70.6	70.4	10.4	56.5	46.1	0.42 (0.20)
2005–2006	83.6	62.1	7.7	76.7	60.8	0.63 (0.25)
*p-value*	*0*.*12*	*0*.*53*	*0*.*12*	*0*.*005*	*0*.*02*	*0*.*006*
Evolution between first and last progress report						
First progress report	64.3	87.5	12.4	55.1	45.7	0.37 (0.24)
Last progress report	86.0	91.3	8.1	67.1	56.5	0.57 (0.28)
*p-value*	*<* .*0001*	*0*.*08*	*0*.*07*	*0*.*004*	*0*.*002*	*<* .*0001*

Viramune Donation Programme, sub-Saharan Africa, 2001–2011 (results are expressed in percentages, except for NCR). SD = standard deviation. NGOs: non-governmental organisations, IAs: international agencies; sd-NVP: single-dose nevirapine; NCR: observed number of women receiving NVP divided by number of women who should have received NVP (number of women in ANC visits x observed HIV seroprevalence).

*of those tested

^†^of those HIV+ counselled.

For each of the PMTCT services indicators assessed, activity increased between the first and the last progress report. The mean number of women attending an ANC, pre-counseled and tested, post-test counseled, and receiving sd-NVP increased by 9,125 women per program-month (SD = 14,286), 6,699 per program-month (SD = 13,252), 4,689 per program-month (SD = 9,855), and 353 per program-month (SD = 900), respectively. The mean number of infants receiving sd-NVP increased by 269 per program-month (SD = 686), and the mean number of new PMTCT sites opened increased by 11.4 sites per program-month (SD = 32.1).

**Fig 1:**
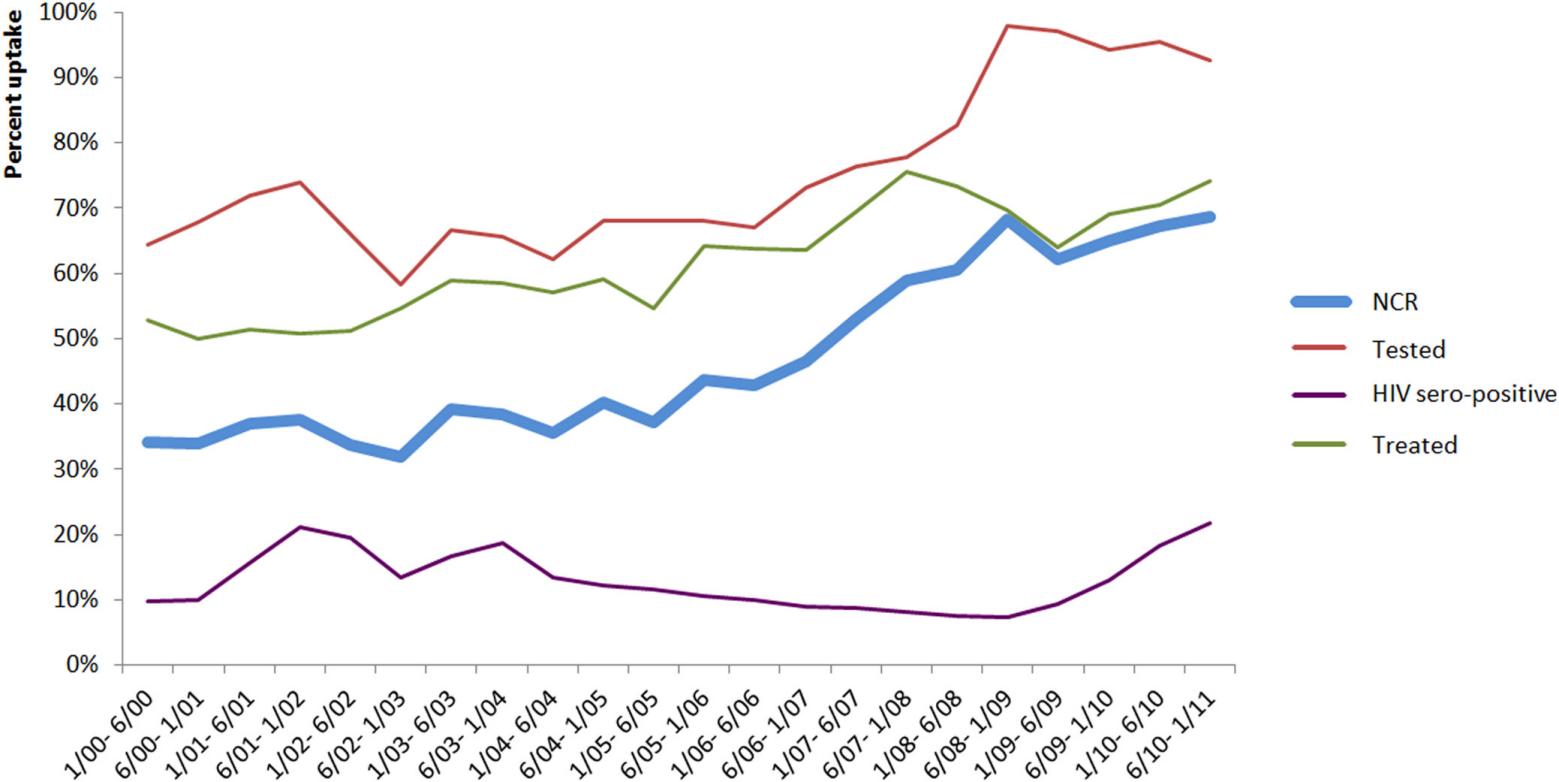
Evolution of proportion of tested women, HIV-positive and treated by Nevirapine (Sd-NVP) and Nevirapine Coverage Ratio (NCR) by semester (N = 64 programs). Viramune Donation Programme, sub-Saharan Africa, 2001–2011 (results are expressed in percentages).

Fig 1 shows the evolution of the proportion of women undergoing HIV testing, testing positive, and receiving NVP, as well as the NCR, by semester from 2001 to 2011. Between 2002 and 2009, NCR increased from about 31.2% to about 68.4%. The proportion of women tested for HIV and treated with sd-NVP also increased during the study period (Fig 1).

The results of multivariate analysis of predictive factors associated with NCR are shown in Table 3. Mixed linear regression analysis found that management by an IA, time since program initiation and program initiation in the 2005–2006 period were significant positive predictive factors of increased NCR. The effect of the time period of the first progress report increased from earlier to later periods. A mean of more than 100 women attending an ANC per month and per site was significantly associated with the NCR, with a negative dose-response relationship according to the three categories (Table 3).

**Table 3:** Mixed linear regression analysis of predictive factors associated to the Nevirapine Coverage Ratio (NCR). Viramune Donation Programme, sub-Saharan Africa, 2001–2011 (N = 64 programs) (results are expressed in percentages).

	β coefficient	95% CI	p
Responsible institution			
Government	0 (ref)	-	-
NGO	8.44	-2.60 to 19.48	0.13
IA	20.87	6.29 to 35.45	0.005
			
Number of women attended in ANC (per month/site)			
<30	0 (ref)		
30 to100	-7.53	-15.67 to 0.62	0.07
≥100	-16.42	-26.63 to -6.21	0.002
			
Total number of sites			
<20	0 (ref)		
20 to100	5.21	-3.62 to 14.04	0.25
≥100	4.42	-5.99 to 14.84	0.40
			
Number of new sites	-0.01	-0.05 to 0.02	0.42
			
HIV prevalence (%)	-0.10	-15.78 to 15.58	0.990
			
Time since program starting (per year)	3.26	1.46 to 5.07	0.0001
			
Time period of the first progress report			
2001–2002	0 (ref)		
2003–2004	1.26	-11.47 to 14.00	0.85
2005–2006	26.29	12.02 to 40.55	0.0001

NCR: observed number of women receiving NVP divided by number of women who should have received NVP (number of women in ANC visits x observed HIV seroprevalence); ß coefficients and their 95% confidence intervals indicating the difference in NCR (0–100)

## Discussion

This large longitudinal study comprises a diverse set of 64 PMTCT programs in 25 sub-Saharan African countries with median follow up of 45.8 months. Over the course of the VDP, the NCR increased by 3.26% per year-program, the mean number of women attending an ANC and undergoing pre-test counseling, HIV testing, and post-test counseling increased significantly, as did the number of women and infants receiving sd-NVP.

The sd-NVP coverage was higher for programs that were initiated at later dates, as shown by a significantly higher NCR for programs starting in 2005–2006 compared with earlier time points. This suggests that PMTCT programs initiated later may have learned from the experiences of programs already implemented. External factors may also have increased overall awareness of and demand for such programs and driven additional women to attend an ANC, including ANCs participating in the VDP. These factors include an overall increase in access to healthcare, availability of healthcare resources and tools, and lessons learned from a variety of PMTCT and other access programs including those implemented by the Elizabeth Glaser Pediatric AIDS Foundation, the President’s Emergency Plan for AIDS Relief (PEPFAR), and donations from various governmental and philanthropic organizations [24].

The overall NCR over the course of this study was 52%, and NCR increased throughout the study period, especially after 2006. The maximum NCR of 66% in 2009 is quite similar to the 59% (CI = 53%-66%) coverage of PMTCT services estimated by UNAIDS [26]. Our findings suggest that large-scale, sd-NVP-based perinatal PMTCT programs are routinely feasible in resource-constrained countries, especially when drugs are donated and most of the programs are specifically developed for PMTCT activities and supported financially.

The NCR was higher for programs managed by NGOs and IAs compared with those managed by governmental institutions. The type of managing organization appears to be a significant predictive factor for increased NCR. Several factors may account for this observation. First, the mean number of women attending an ANC was highest for sites managed by governmental organizations compared with NGOs or IAs, while programs with more than 100 women attending an ANC per month had significantly lower NCR than programs seeing fewer women each month. This suggests that the governmental sites may have had larger infrastructure or logistics challenges due to their need to meet greater demand than sites managed by other types of organizations. Consequently, the reduced NCR associated with government-run programs may be associated with the availability of resources (i.e. for international agencies) rather than technical competency. Second, NGOs and IAs are usually smaller than government organizations, with fewer key decision makers and layers of regulations, which may allow them to move more quickly in developing and mobilizing resources, as well as implementing and modifying programs. Thus, increased NCR for these organizations may be independent of the ability of specific types of institutions to execute PMTCT programs effectively. Moreover, because NGOs and IAs were permitted to participate in VDP with the approval of the government(s) in which their programs were to be conducted, it is likely that these organizations had governmental support that served as an additional asset in their efforts to deploy programs effectively.

The rapid increase in the number of new PMTCT sites added per month between the first and last progress report suggests that programs had the operational capacity to develop and expand access to their PMTCT services. Strengthening operational capacity will be important for building PMTCT strategies that are integrated with routine care of women and children and HIV care and treatment programs, and for providing a suite of services and innovative approaches that can reduce MTCT [27–32]. However, our finding that a mean of more than 100 women attending an ANC per month per site was negatively and significantly associated with the NCR, and that NCR decreased with a dose-response relationship as the number of women attended in ANC per 1000/month/site increased, suggests that expanding the breadth of services that an ANC provides should be done cautiously and with a goal of ensuring sufficient infrastructure and personnel resources to ensure success. The use of quality improvement programs, mobile-phone-based outreach and follow-up systems, and strategies for increasing healthcare workers’ motivation may allow increasing numbers of women to be seen at sites of integrated PMTCT programs providing comprehensive female, maternal, and child healthcare and HIV treatment and prevention services without negatively impacting the PMTCT access [8, 14, 33–35]. The 3.26% increase in NCR per year-program suggests that programs can continue to improve performance over time.

There is great potential for PMTCT programs to have a significant impact on reducing the burden of HIV/AIDS and improving health outcomes, especially for children, in sub-Saharan Africa [1, 24, 33]. Identifying criteria that predict success of such programs is important for implementing future programs for HIV care and may also be relevant to chronic diseases, such cancer, cardiovascular diseases, or diabetes [24].

Our study has several limitations that should be noted. Longitudinal data collection and monitoring were difficult over the life of the programs. However, the VDP program manager regularly assessed the reporting data system, and reporting requirements were communicated to the program manager at the time the program was included in the VDP. Data were reported as expected and consistent with requirements, adding to the strength of the data set. Another limitation is that we could not examine potential factors that might explain observed patterns, such as variables related to socioeconomic resources, rural/urban setting, type of health facility (dispensary or health center), access to health care, and quality of care. Funding from international programs and initiatives also could have been potential factors leading to better performance over time. A third limitation derives from the fact that we cannot confirm that our results are representative of all PMTCT programs implemented in sub-Saharan Africa, particularly those based on different ARV regimens. We were also not able to determine if a pregnant woman actually took the sd-NVP dose she was given in PMTCT services, or if she administered the dose to her newborn infant because we lacked the ability to directly measure the drug compounds contained in cord blood samples. Identification of the date of program initiation as a significant predictor of NCR is also a limitation given that a past date cannot be used to predict performance of programs initiated since that time. However, as noted above, several factors associated with the 2005–2006 period (increased awareness of PMCTC, additional resources and funding for PMTCT programs, and lessons learned from earlier PMTCT initiatives) could be relevant for the design and implementation of future PMTCT programs. Finally, our findings rely on data covering an early era in large-scale PMTCT programs, when simpler antiretroviral regimens were used as opposed to the multi-drug regimens currently recommended. We believe that it is important to document the barriers encountered in interventions as simple as those based on single-dose NVP, because it is likely that such factors may also apply to the more complex interventions of today. These factors include the type of responsible institution, the overall size of the program in terms of number of sites, the monthly number of women attending ANC, and the related logistics challenges associated with these factors. Despite these limitations, we believe that the factors identified as predictors of program performance may help to inform the development of additional public health programs that can help reduce MTCT of HIV in resource-constrained environments such as sub-Saharan African countries.

## Conclusion

This study identified a number of predictive factors that contribute to uptake of PMTCT coverage in sub-Saharan Africa countries and highlights the challenges and potential benefits of scaling up PMTCT programs in sub-Saharan Africa countries. Additional insight into programmatic factors associated with performance is essential for further expansion and scale-up of PMTCT in resources-constrained countries. Performance may be better for PMTCT programs with a small or medium number of women attended in ANC. While additional research is needed and many public health challenges remain, overall strengthening of heath systems, increasing educational strategies, enhanced counseling, and community support have the potential to improve access to PMTCT services in sub-Saharan Africa.
